# Workforce and Task Sharing of Nurses in the Japanese Intensive Care Unit-Cross-Sectional Postal Survey

**DOI:** 10.3390/healthcare9081017

**Published:** 2021-08-07

**Authors:** Takeshi Unoki, Yusuke Kawai, Miya Hamamoto, Mitsuhiro Tamoto, Takeharu Miyamoto, Hideaki Sakuramoto, Yumi Ito, Etsuko Moro, Junko Tatsuno, Osamu Nishida

**Affiliations:** 1 Department of Acute and Critical Care Nursing, School of Nursing, Sapporo City University, Kita 11 Nishi 13, Chuo-ku, Sapporo 060-0011, Japan; 2 Department of Nursing, Fujita Health University Hospital, 1-98 Dengakugakubo, Kutsukake, Toyoake 470-1192, Japan; kawai@kkh.biglobe.ne.jp; 3 Intensive Care Unit, Tosei General Hospital, 160 Nishioiwake, Seto 489-8642, Japan; ichochan13@yahoo.co.jp; 4 Intensive Care Unit, Kyoto University Hospital, 54 Kawaharacho, Shogoin, Sakyo-ku, Kyoto 606-8507, Japan; tamoto@kuhp.kyoto-u.ac.jp; 5 Department of Nursing, Faculty of Health Sciences, Junshin Gakuen University, 1-1-1 Chikushigaoka, Minami-ku, Fukuoka 815-1510, Japan; miyamoto.t@junshin-u.ac.jp; 6 Department of Adult Health Nursing, College of Nursing, Ibaraki Christian University, 6-11 Omika, Hitachi 319-1295, Japan; gongehead@yahoo.co.jp; 7 Department of Nursing, Faculty of Health Sciences, Kyorin University, 6-20-2 Shinkawa, Mitaka 181-8611, Japan; yumito@ks.kyorin-u.ac.jp; 8 Intensive Care Unit, Jichi Medical University Hospital, 3311-1 Yakushiji, Shimotsuke 329-0498, Japan; e-moro@jichi.ac.jp; 9 Department of Nursing, Kokura Memorial Hospital, 3-2-1 Asano, Kokurakita-ku, Kitakyushu 802-8555, Japan; tatsuno3@icloud.com; 10 Department of Anesthesiology and Critical Care Medicine, School of Medicine, Fujita Health University, 1-98 Dengakugakubo, Kutsukake, Toyoake 470-1192, Japan; nishida@fujita-hu.ac.jp

**Keywords:** intensive care units, workforce, mechanical ventilation, extracorporeal membrane oxygenation

## Abstract

This study aimed to estimate the number of nurses who independently care for patients with severe respiratory failure receiving mechanical ventilation (MV) or veno-venous extracorporeal membrane oxygenation (VV-ECMO). Additionally, the study analyzed the actual role of nurses in the treatment of patients with MV and VV-ECMO. We performed a cross-sectional study using postal questionnaire surveys. The study included 725 Japanese intensive care units (ICUs). Data were analyzed using descriptive statistics. Among the 725 ICUs, we obtained 302 responses (41.7%) and analyzed 282 responses. The median number of nurses per bed was 3.25. The median proportion of nurses who independently cared for patients with MV was 60% (IQR: 42.3–77.3). The median proportion of nurses who independently cared for patients with VV-ECMO was 46.9 (35.7–63.3%) in the ICUs that had experience with VV-ECMO use. With regard to task-sharing, 33.8% of ICUs and nurses did not facilitate weaning from MV. Nurses always titrated sedative dosage in 44.5% of ICUs. Nurse staffing might be inadequate in all ICUs, especially for the management of patients with severe respiratory failure. The proportion of competent nurses to care for severe respiratory failure in ICUs should be considered when determining the workforce of nurses.

## 1. Introduction

The coronavirus disease (COVID-19) has been spreading globally since January 2020. The increase in the number of infections has resulted in an increasing number of severely ill patients. Critically ill COVID-19 patients require mechanical ventilation (MV), as they usually present with severe respiratory failure, along with veno-venous extracorporeal membrane oxygenation (VV-ECMO), which results in a heavy burden on intensive care units (ICUs) [1], and the surge in the number of infected patients has overburdened ICUs. Therefore, nurses without ICU experience have had to engage in ICU nursing, which has exposed them to high stress and has necessitated their engagement in unfamiliar nursing care tasks in the ICU [2]. In order to avoid such disastrous events, it is necessary to plan strategies in advance to ensure that the ICU will be able to handle a heavy burden in a strategically planned manner.

The ICUs that meet Japan’s Ministry of Health, Labor and Welfare (MHLW)-specified criteria are divided into eight types for reimbursement purposes. Among them, six types are described below, wherein a ratio of more than 0.5 nurses per patient is to be maintained at all times. ICU types 1 and 2 must include at least two physicians with ICU experience (not necessarily intensivists), as well as nurses with specialized training. ICU types 3 and 4 have the same nursing arrangements but without the requirement for physicians or specialized nurses. Emergency-specialized ICU types 2 and 4 that are located at emergency medical centers have the same requirements for nursing staff, but there are no requirements for physicians or specialized nurses [3].

A 2018 Japanese governmental report indicates that there were 785 units, with approximately 24,000 nurses working in ICUs [4]. COVID-19 can cause severe respiratory failure, and patients in respiratory distress are admitted to the ICU. Critical care nurses provide holistic nursing, including the management of complicated devices such as MV and VV-ECMOs. Therefore, a certain level of experience and training are essential for critical care nurses [5]. However, so far, it is unclear as to how many critical care nurses, who can tend to patients with severe respiratory failure with MV and/or VV-ECMO work in these ICUs. Therefore, it is important to determine the number of nurses who tend to patients with severe respiratory failure in order to plan for future crises, including pandemics.

When considering the required number of nurses, awareness of the task-sharing or task-shifting situations is essential. Intensive care is provided by a multi-professional team; however, the role of nursing professionals varies among different facilities. Generally, the role of nurses tends to expand in the ICU. For example, nurse-based weaning from MV based on a protocol decreased the duration of MV [6]. Additionally, the dose titration of sedatives and analgesic agents by nurses decreases the duration of MV [7]. We speculate that the responsibility for weaning from MV and for other procedures was shared with other professionals, such as the physician, physiotherapist, or clinical engineer. However, there are no data with respect to the actual role of nurses working in ICUs in Japan.

This study aimed to estimate the proportion of nurses who could care independently for patients with severe respiratory failure and to analyze the actual role of nurses in providing clinical care for such patients. We defined a “nurse who cares independently” as a nurse who provided standard or skilled care without follow-up from senior nursing staff.

## 2. Materials and Methods

### 2.1. Study Design

A cross-sectional study on Japanese ICUs was undertaken using a postal questionnaire survey, wherein the survey questionnaire sets were sent to each ICU, and the data obtained were analyzed using descriptive statistics. Some of the data pertaining to COVID-19 have been previously published due to the high relevance and importance of the preliminary results [8].

### 2.2. Participants and Setting

We included all ICUs that were more than 0.5 nurses per patient at all times in Japan. We excluded ICUs that were clearly designated for pediatric care based on a review of the names of the ICUs. We sent out a questionnaire for each ICU; 725 ICUs met our criteria, and we sent 725 questionnaires to them on 20 October 2020 and collected the questionnaires on 15 November 2020. We included a provision for a delayed survey response until 10 January 2021.

### 2.3. Variables

The research team consisted of 5 critical care nurses and 4 nurse researchers with ICU practice experience who developed a questionnaire based on discussion. The survey set included queries to obtain details of the ICU and hospital characteristics. Additionally, we obtained the number of nurses working in ICUs, new graduates, and nurses who independently provided nursing care for patients with severe respiratory failure with MV and VV-ECMO. In total, there were 52 questions. Respondents described the number of nurses as of January 2021, just before the pandemic spread to Japan. The actual work-sharing situation of the nurse to other professionals, such as the physician, physiotherapist, or clinical engineer, according to the use of the equipment and medical management was described (i.e., MV maintenance, titration of medication dose according to the protocol, and priming of the circuit for VV-ECMO). Respondents were asked to rate their implementation status using a five-point scale, with responses ranging from “always” to “never.” “Always” means that a nurse always did the task, and “never” means that a nurse never undertook the task.

### 2.4. Measurement

Only nurse managers, certified nurse specialists, and certified nurses filled out the survey. All data were extracted from the returned survey sheet, except for the number of hospital beds.

### 2.5. Data Analysis

A professional survey company extracted the data and verified the data points. Some datasets were incomplete due to missing values. Thus, the number of denominators varied in each analysis. On visual inspection, impossible answers were excluded based on standards that were defined based on a discussion of the research team. For example, minimum staffing requirements were needed to ensure that a nurse-to-patient ratio of 1:2 was maintained. If they were not met, they were treated as missing values. Descriptive statistical analyses were conducted. Continuous and ordinal variables are expressed as the median and interquartile range. Categorical data are expressed as numbers and percentages unless otherwise specified. We used the Wilcoxon-log-rank test for continuous and ordinal variables and the chi-square test for two or more categorical variables, to assess for differences. Analysis was conducted in Stata/IC 16.1 (Statacorp, College Station, TX, USA) and R 4.0.2 (R foundation). We considered a *p*-value of less than 0.05 to be statistically significant.

### 2.6. Ethical Considerations

This study was approved by the research ethics review committee of Sapporo City University (No. 2008-1), Sapporo, Japan. The survey instructions clearly stated that study participation was voluntary. The return of the survey form was considered as consent.

## 3. Results

**Figure 1:**
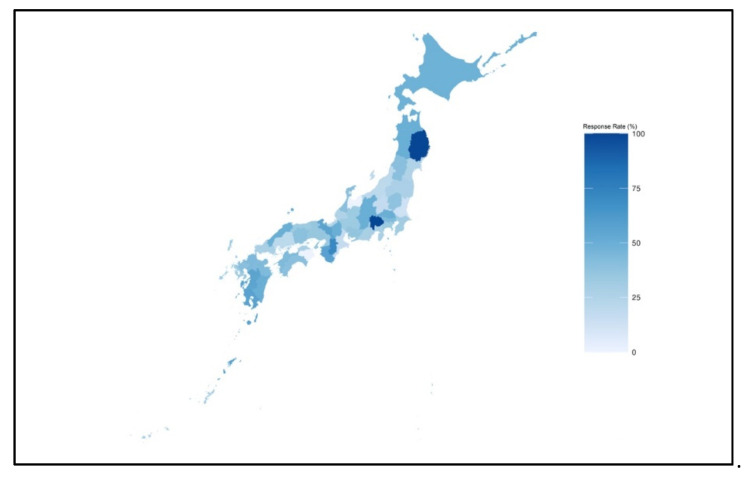
The response rate of the postal survey in each prefecture.

Of the 725 questionnaires sent out, 302 were returned. The response rate was 41.7%. We excluded 20 responses (6.6% of the responses), as 7 units focused on pediatric patients and 11 were ineligible. Two surveys were excluded as one was not filled out by a predefined person, whereas another contained invalid answers. Consequently, 282 responses from ICUs were analyzed. Concerning geographical distribution, Figure 1 indicates the proportion of ICUs, by prefecture, that were mailed the study questionnaires and the ICUs that responded.

Dark blue indicates a high response rate at the prefecture level.

The characteristics of the responding ICUs are shown in Table 1. The median numbers of ICU beds and nurses per bed were 8 (6–12) and 3.25 (2.85–3.75), respectively.

The number of nurses who were independently providing care for patients with MV and/or VV-ECMO is shown in Table 2. The median proportion of nurses who independently cared for patients with MV was 60% (42.3–77.3%). In total, 133 (47.1%) ICUs had experience with VV-ECMO. In these experienced ICUs, the median proportion of the number of nurses who independently cared for patients with VV-ECMO was 46.9% (35.7–63.3%; Table 2).

**Figure 2:**
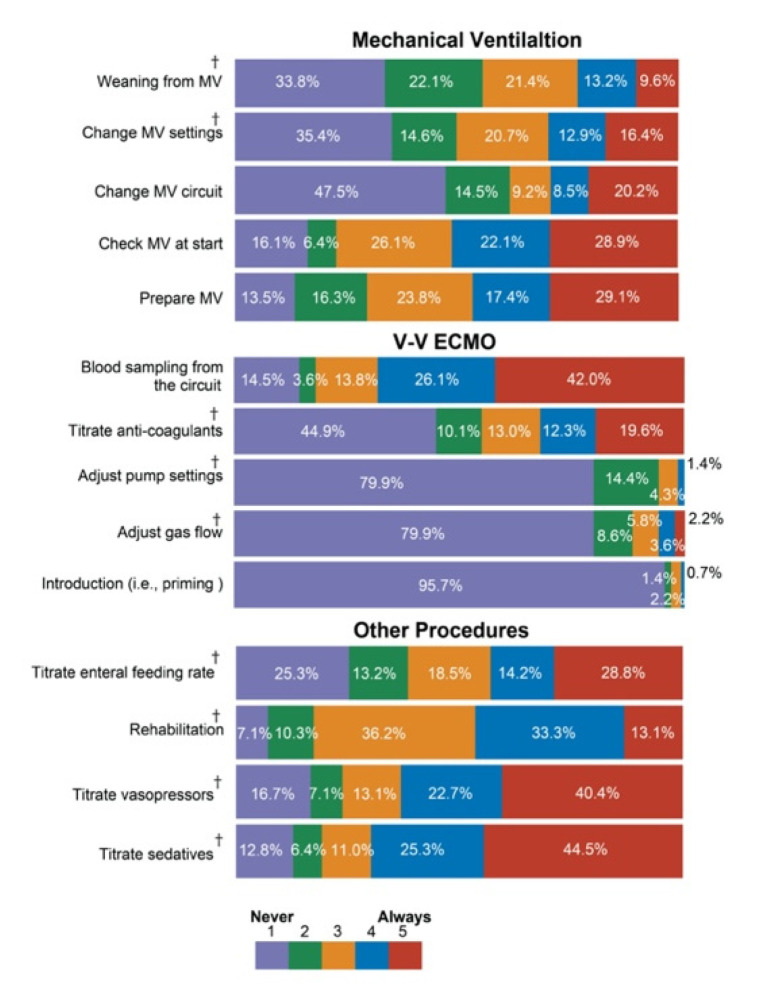
Nursing activities for specific procedures.Note: “Always” means that the nurse always undertook the procedure. “Never” means that the nurse never undertook the procedure and that other professionals, such as physicians, undertook the task. Missing data were included in this analysis. Abbreviations: MV, mechanical ventilator; VV-ECMO, veno-venous extracorporeal membrane oxygenation; ^†^ under a pre-prescribed protocol.

Figure 2 indicates the actual task-sharing status. In 33.8% of the ICUs, nurses never facilitated weaning from MV. In most of the ICUs, nurses never undertook any activity pertaining to VV-ECMO except for blood sampling from the circuit and dose titration of anticoagulant agents. In 44.5% of the ICUs, nurses always titrated the dose of sedatives. Similarly, in 40.4% of the ICUs, the nurse always titrated the dose of vasoactive agents.

## 4. Discussion

This is the first survey to estimate the actual number of nurses who independently provided care for patients with severe respiratory failure. To our knowledge, this is the first study to clarify the actual task-sharing of nurses with other medical professionals, such as physician, physiotherapist, or clinical engineers, in the Japanese ICU. From the geographic distribution and proportion of ICUs from university hospitals, our findings did not indicate significant selection bias and were representative of Japanese ICUs.

The ICUs may not have a sufficient number of nurses per bed, with a median number of 3.25. However, the median numbers from Australia and New Zealand were 4.7 ± 1.2 and 4.2 ± 1.4 nurses per bed, respectively [9]. In Japan, the number of nurses per patient at all times is more than 0.5, as determined by the government. Maintaining a nurse-to-patient ratio of 1:1 or 1:2 for critically ill patients in each shift, such as the day shift, is determined by the facility. Perhaps, a small number of nurses per bed means fewer nurses during the day to maintain a 1:2 nurse-to-patient ratio. For reimbursement purposes, a 1:2 nurse-to-patient ratio during the day is acceptable, as there is no regulation that determines the number of patients assigned per nurse based on disease severity. Internationally, the nurse-to-patient ratio ranges from 1:1 to 1:2, as determined by the acuity scoring system in Massachusetts [10]. The majority of ICUs in the UK had a nurse-to-patient ratio of 1:1 [11]. A nurse was assigned for every patient with invasive MV in Australia and New Zealand [9]. Recently, it was suggested that a nurse-to-patient ratio of 1:1.5 in the ICU was associated with reduced mortality [12,13]. It is difficult to generalize the situation because of the varying systems and different populations entering the ICU globally. However, we can potentially consider a system that requires nurses to be assigned according to the disease severity. The adequate assignment of patients in the ICU is not necessarily determined by the amount of assistance or monitoring devices. Alongside these factors, we analyzed whether frequent observation is an important prerequisite. There is a potential need to reconsider the adequate nurse-to-patient ratio in Japanese ICUs.

We emphasized that not all critical care nurses were able to independently care for patients with severe respiratory failure. Nursing management for this specific population is challenging [14]. To our knowledge, there are no formal training courses in Japan other than those for certified nurses and certified nurse specialists; however, certified courses take 0.5–2 years with full-time course requirements. Therefore, not many nurses take up the courses. To facilitate the competency of care for critically ill patients, such as those with severe respiratory failure, formal and standardized courses delivered via flexible modes, such as e-learning, are needed [15]. The development of a standardized course and certification should be considered.

Approximately half of the ICUs had experience with VV-ECMO use. Approximately 46% of nurses in the ICU were able to care for patients with severe respiratory failure with VV-ECMO. Notably, this information is only available for facilities that have experience with VV-ECMO. We noted that the number of cases receiving VV-ECMO in an ICU was very low, which indicated that ICU treatment with VV-ECMO was not centralized. VV-ECMO is complex [16,17] and requires experience; thus, for sufficient caseload and maintaining competence, ICUs using VV-ECMO should be centralized [18].

The number of intensivists in Japan is very low [19], and they are therefore often overburdened. Promotion of the work-style reform of physicians is among the most important medical policies of the Japanese government. Task-shifting in ICUs may contribute to reducing the burden on intensivists. Especially in VV-ECMO, nurses did not perform introduction, including priming and maintenance. Clinical engineers should perform these tasks to ensure safety [20]. Conversely, nurses are often involved in the titration of sedatives, analgesics, and vasopressors. As described above, previous studies have suggested that nurse-led sedation protocols lead to favorable outcomes [21]. From a patient-centered point of view, these contribute to optimal outcomes. It is important to promote task-sharing to reduce the burden on intensivists and improve the outcomes of critically ill patients. However, systematic continuous education of administration for these drugs is required [22]. Further studies are required to determine whether critical care nurses receive adequate education regarding sedatives and analgesics and others. Additionally, studies examining the association between adequate education and patients’ outcome are warranted.

## 5. Limitations

As the response rate was approximately 40%, there was potential for selection bias. However, ICUs that responded had a broad range of characteristics, including different geographical areas and various numbers of ICU beds, and were within different types of hospitals (university versus non-university). Therefore, our findings reflect the current clinical practices of various ICUs in Japan.

## 6. Conclusions

Our findings suggest that approximately 60% of nurses independently care for patients with severe respiratory failure receiving MV. Additionally, few nurses independently care for patients with severe respiratory failure who are treated with VV-ECMO. Along with experience, continuous, standardized education is required to increase the number of nurses who can care for patients with severe respiratory failure.

There is evidence that task sharing or shifting that achieves optimal outcomes for patients should be facilitated. For safety, adequate education for critical care nurses will be required.

## Figures and Tables

**Table 1:** Characteristics of the intensive care units included in the analysis.

Variables	Values
**Type of hospital (%), *n* = 282**	
University hospital	73 (25.9%)
Missing	5 (1.8%)
**Type of ICU based on medical fee (%), *n* = 282**	
ICU type 1, 2	108 (38.3%)
ICU type 3, 4	130 (46.1%)
Emergency ICU 2, 4	44 (15.6%)
**Physician staffing (%), *n* = 282**	
Closed ICU ^†^	21 (7.4%)
Mandatory intensivist consultation	156 (55.3%)
Elective intensivist consultation	48 (17%)
No intensivist	56 (19.9%)
Missing	1 (0.4%)
**ICU beds and Nurse staffing**	
Number of ICU beds, median [IQR], *n* = 282	8 (6–12)
Number of ICU nurses, median [IQR], *n* = 265	30 (22–38)
Proportion for number of nurses with <1 year of ICU experience, median [IQR], *n* = 263	15.6% (11.1–22.6)
No certified nurse or certified nurse specialist, *n* (%), *n* = 282	41 (14.5%)

^†^ Intensivists manage all ICU patients. Abbreviations: VV-ECMO, Veno-Venous extracorporeal membrane oxygenation; IQR, interquartile range; ICU, intensive care unit.

**Table 2:** Number of nurses independently providing care for mechanical ventilation and VV-ECMO for patients with severe acute respiratory failure.

Variables	Values
**Mechanical Ventilation for Severe Respiratory Failure**	
Number of nurses independently caring for the specified population in a unit, median (IQR), *n* = 259 ICUs	17 (12–23)
Proportion of nurses independently caring for the specified population in a unit, median (IQR), *n* = 259 ICUs	60 (42.3–77.3)
**VV-ECMO for Severe Respiratory Failure**	
**Characteristics of ICU using VV-ECMO, *n* = 133 ICUs**	
University hospital, *n* (%), *n* = 129 ICUs ^†^	48 (37%)
Number of cases with VV-ECMO per a year, median [IQR], *n* = 133	2 (1–5)
**Nurses independently providing care for VV-ECMO, *n* = 133 ICUs**	
Number of nurses independently caring for the specified population in a unit, median (IQR), *n* = 117 ICUs *	15 (10–21)
Proportion of nurses independently caring for the specified population in a unit, median (IQR), *n* = 117 ICUs *	46.9 (35.7–63.3)

Abbreviations: VV-ECMO, Veno-Veno extracorporeal membrane oxygenation: IQR, interquartile range. ^†^ Responses from 4 ICUs were missing for this point. * Responses from 16 ICUs were missing for this point.

## Data Availability

All data from this research have been included within the manuscript.
